# Quantitative Imaging of Hypoxic CAIX-Positive Tumor Areas with Low Immune Cell Infiltration in Syngeneic Mouse Tumor Models

**DOI:** 10.1021/acs.molpharmaceut.3c00045

**Published:** 2023-03-07

**Authors:** Daan F. Boreel, Paul N. Span, Annemarie Kip, Milou Boswinkel, Johannes P. W. Peters, Gosse J. Adema, Johan Bussink, Sandra Heskamp

**Affiliations:** † Radiotherapy and OncoImmunology Laboratory, Radiation Oncology, Radboud University Medical Center, Geert Grooteplein Zuid 32, 6525GA Nijmegen, The Netherlands; ‡ Department of Medical Imaging, Radboud University Medical Center, Geert Grooteplein Zuid 10, 6525GA Nijmegen, The Netherlands

**Keywords:** carbonic anhydrase IX, hypoxia, animal imaging, immunology, tumor microenvironment

## Abstract

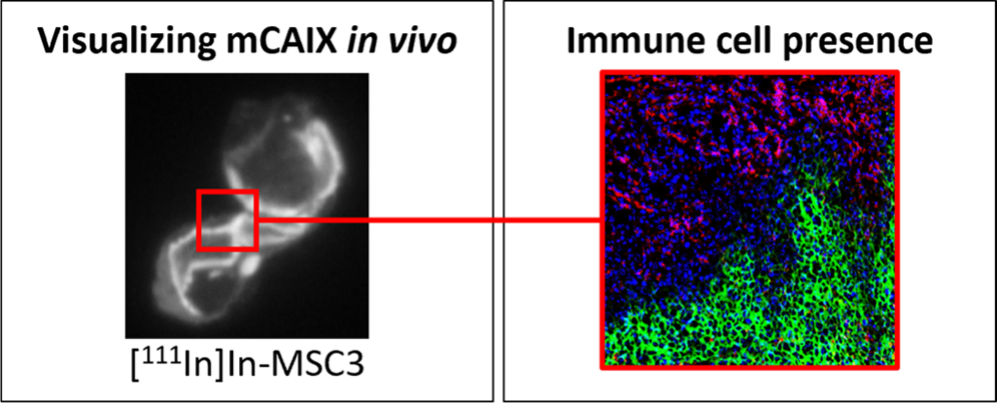

Limited diffusion of oxygen in combination with increased oxygen consumption leads to chronic hypoxia in most solid malignancies. This scarcity of oxygen is known to induce radioresistance and leads to an immunosuppressive microenvironment. Carbonic anhydrase IX (CAIX) is an enzyme functioning as a catalyzer for acid export in hypoxic cells and is an endogenous biomarker for chronic hypoxia. The aim of this study is to develop a radiolabeled antibody that recognizes murine CAIX to visualize chronic hypoxia in syngeneic tumor models and to study the immune cell population in these hypoxic areas. An anti-mCAIX antibody (MSC3) was conjugated to diethylenetriaminepentaacetic acid (DTPA) and radiolabeled with indium-111 (^111^In). CAIX expression on murine tumor cells was determined using flow cytometry, and *in vitro* affinity of [^111^In]In-MSC3 was analyzed in a competitive binding assay. *Ex vivo* biodistribution studies were performed to determine *in vivo* radiotracer distribution. CAIX^+^ tumor fractions were determined by mCAIX microSPECT/CT, and the tumor microenvironment was analyzed using immunohistochemistry and autoradiography. We showed that [^111^In]In-MSC3 binds to CAIX-expressing (CAIX^+^) murine cells *in vitro* and accumulates in CAIX^+^ areas *in vivo*. We optimized the use of [^111^In]In-MSC3 for preclinical imaging such that it can be applied in syngeneic mouse models and showed that we can quantitatively distinguish between tumor models with varying CAIX^+^ fractions by *ex vivo* analyses and *in vivo* mCAIX microSPECT/CT. Analysis of the tumor microenvironment identified these CAIX^+^ areas as less infiltrated by immune cells. Together these data demonstrate that mCAIX microSPECT/CT is a sensitive technique to visualize hypoxic CAIX^+^ tumor areas that exhibit reduced infiltration of immune cells in syngeneic mouse models. In the future, this technique may enable visualization of CAIX expression before or during hypoxia-targeted or hypoxia-reducing treatments. Thereby, it will help optimize immuno- and radiotherapy efficacy in translationally relevant syngeneic mouse tumor models.

## Introduction

Hypoxia is a common feature of many solid malignancies and has been linked to radiotherapy resistance, increased metastatic potential, metabolic rewiring, and immune suppression.^1−3^ Hypoxia can be acute or transient due to temporal occlusions of leaky tumor vasculature.^4^ However, a substantial proportion of hypoxia occurs when the oxygen demand exceeds supply, leading to hypoxia at an increased distance from vessels, referred to as chronic or diffusion-limited hypoxia.^5^ These chronic hypoxic areas are specifically associated with poor infiltration of effector immune cells, resulting in an immunosuppressive microenvironment.^3,6^

Tumor hypoxia is a prognostic biomarker that can impact the therapy outcome of cancer patients.^7^ Visualization and quantification of tumor hypoxic fractions will have implications in therapy planning and adaptation for patients with hypoxic tumors, for instance, in the implementation of radiotherapy planning based on radiotracer uptake, hypoxia-activated therapies, and hypoxia-reducing therapies.^8−11^ There is increasing evidence that novel immunotherapies are less effective in the hypoxic tumor microenvironment, highlighting the importance of hypoxia assessment even more.^12−14^ In recent years, the immunological effects of radiotherapy have become more and more evident, and radio–immunotherapy combinations have gained a lot of interest after the completion of several trials with positive outcomes.^15−17^ To study the effects of chronic hypoxia and hypoxia modification on these combination therapies in preclinical models, a method to visualize chronic hypoxia in immunocompetent mice is required.

Below an oxygen concentration of 2%, the hypoxia-inducible factor (HIF-1α) is stabilized and regulates several pathways in the adaptation to hypoxia.^18,19^ This includes the upregulated expression of carbonic anhydrase IX (CAIX), a transmembrane enzyme regulating intracellular pH.^20^ CAIX has been validated as a prognostic biomarker in several cancers, but its potential predictive value in the context of radio- and immunotherapy makes it an even more interesting imaging target.^21,22^ Existing tracers to image hypoxia, like [^18^F]FAZA and [^18^F]FMISO, function by the entrapment of 2-nitroimidazoles in hypoxic areas,^23^ thereby visualizing both chronic and acute hypoxia. In contrast, CAIX is an endogenous hypoxia marker that, due to its long half-life (38 h), is better suited to visualize chronic hypoxia.^24,25^ CAIX-expressing cancer cells represent a population adapted to chronic hypoxia and are, therefore, highly relevant in the context of cancer immunology research.^26^ Therefore, we pose CAIX as a suitable target for functional imaging of cancer cells that have adjusted to chronic hypoxic conditions. Radiolabeled tracers to image CAIX in human xenograft models have been studied in the past, but radiotracers recognizing murine CAIX, necessary to study mice with an intact immune system, are not available yet.^27^

The aim of this study was to develop an antibody-based imaging tracer for quantitative and noninvasive assessment of CAIX expression in syngeneic mouse tumor models and to correlate tracer uptake to CAIX expression, hypoxia, and the immunological tumor microenvironment.

## Methods

### Cell Culture

B16ova murine melanoma cells (provided by Dr. K.L. Rock, Dana-Farber Cancer Institute, Boston^28^) were cultured in MEM with 5% fetal calf serum (FCS), 2% sodium bicarbonate, 1.5% MEM vitamins, 1% sodium pyruvate, 1% nonessential amino acids, 1% antibiotic/antimycotic, 0.1% β-mercaptoethanol, 1 mg/mL G418, and 60 μg/mL hygromycin (all Gibco). B16ova cells are a B16 variant transfected with ovalbumin that exhibit a different pattern of immune cell infiltration.^29,30^ B16F1 murine melanoma (provided by Dr. M. Schreurs, Radboudumc, Nijmegen) and LLC1 murine lung carcinoma cells (ATCC) were cultured in DMEM Glutamax with 10% FCS, 1% sodium pyruvate, and 1% penicillin–streptomycin (all Gibco). MOC1 and MOC2 (provided by R. Uppaluri, Dana-Farber Cancer Institute, Boston) murine oral squamous cell carcinoma cells were cultured in 2:3 IMDM, 1:3 Ham’s F-12 nutrient mix with 5% FCS, 1% penicillin–streptomycin (all Gibco), 5 ng/mL EGF (EMD Millipore), 40 ng/mL hydrocortisone, and 5 μg/mL insulin (both Sigma Aldrich). For animal experiments, the *in vivo* passaged MOC1-derived clone 3D5 that showed improved tumor take was used. MC38 (Kerafast) murine colon carcinoma cells were cultured in DMEM Glutamax with 10% FCS, 1% sodium pyruvate, 1% nonessential amino acids, and 1% penicillin–streptomycin (all Gibco). GL261 murine glioblastoma cells (provided by U. Herrlinger, University of Bonn, Bonn) were cultured in IMDM, 10% FCS, 0.1% β-mercaptoethanol, and 1% penicillin–streptomycin. SK-RC-52 human clear cell renal cell carcinoma cells (provided by Memorial Sloan Kettering Cancer Center, New York), which ubiquitously express CAIX independent of oxygenation status, were cultured in RPMI-1640 with 10% FCS and 1% glutamine (all Gibco). All cell culture were performed at 37 °C in 5% CO_2_. For *in vitro* experiments, cells were not passaged more than 15 times post thawing. For tumor cell inoculation in *in vivo* experiments, cells were passaged two times post thawing.

### Conjugation and Radiolabeling

Human IgG1 antimouse CAIX (mCAIX, clone MSC3, Creative Biolabs) (molecular weight: 150 kDa), which reacts with both human and murine CAIX, and human IgG1-irrelevant control antibody (hIgG1, BioXCell) (molecular weight: 150 kDa) were conjugated in a molar ratio of 1:15 with isothiocyanatobenzyl–diethylenetriaminepentaacetic acid (ITC–DTPA, Macrocyclis) in NaHCO_3_ (pH 9.5) for 1 h at room temperature (resulting in a DTPA/antibody ratio of 3.4:1). The conjugated antibody was dialyzed against NH_4_Ac (0.25 M, pH 5.5) to remove the unbound chelator. DTPA-conjugated antibodies were radiolabeled with indium-111 (^111^In, Curium) after adding a twofold volume of 2-(*N*-morpholino)ethanesulfonic acid (0.5 M MES, pH 5.5) buffer for 30 min at RT. Radiolabeling efficiency exceeded 95% in all experiments, as determined by instant thin-layer chromatography on silica gel chromatography strips (ITLC-SG, Agilent Technologies) in 0.1 M sodium citrate buffer (Sigma Aldrich).

### *In Vitro* Assays

Cells were cultured in 6-well plates to confluency. To induce CAIX expression, cells were cultured under hypoxic conditions of 1% O_2_ for 24, 48, or 72 h in a Whitley H35 Workstation (Don Whitley Scientific). The binding of radiolabeled antibodies and 50% inhibitory concentration (IC_50_) were determined as described by Heskamp et al.^31^ The percentage of CAIX^+^ cells was determined by flow cytometry analysis on a FACSCanto II (BD Bioscience). Cells were harvested, and fixable viability dye eFluor 780 (eBioscience, 65-0865-14) was added to distinguish dead and live cells. Cells were stained with Human IgG1 anti-mouse CAIX (MSC3) for 30 min at 4 °C and with goat anti-human Alexa Fluor 488 (Invitrogen, A-11013) for 15 min at 4 °C. Data were analyzed using FlowJo V10.7 (Tree Star).

### Animal Experiments

All animal experiments were conducted in accordance with the principles laid out by the Dutch Act on Animal Experiments (2014) and approved by the Animal Welfare Body of the Radboud University Nijmegen and the Central Authority for Scientific Procedures on Animals. Female C57BL/6 mice (10–12 weeks, Charles River) were inoculated subcutaneously with 1.0 × 10^6^ B16ova or B16F1 in phosphate-buffered saline (PBS) or with 1.0 × 10^6^ MOC1.3D5 cells in 1:3 matrigel/PBS (BD Bioscience) on the right flank. Experiments were started when tumors reached a size of approximately 100 mm^3^.

### CAIX-Specific Accumulation of [^111^In]In-MSC3 in Hypoxic Tumor Regions, Hypoxia, and Perfusion Markers

B16ova-tumor-bearing mice were injected intravenously (i.v.) via the tail vein with 10 μg of (1.2 MBq/μg) [^111^In]In-MSC3 or [^111^In]In-hIgG1. At 72 h post tracer injection, mice were sacrificed, and tumors were collected for immunohistochemistry (IHC) and autoradiography (AR). To mark hypoxia and perfusion in all subsequent experiments, mice were injected with pimonidazole (J. A. Raleigh, Department of Radiation Oncology, University of North Carolina) intraperitoneally 1 h prior to sacrifice, 80 mg/kg, and Hoechst 33342 (Sigma) at 1 min prior to sacrifice, i.v., 15 mg/kg.

### Dose Optimization and Pharmacokinetics of [^111^In]In-MSC3

For antibody dose optimization, four groups of six mice with subcutaneous B16ova tumors received an i.v. injection in the tail vein of 3 μg of (0.33 MBq/μg) [^111^In]In-MSC3 or an i.v. injection with 3 μg of (0.33 MBq/μg) [^111^In]In-MSC3 supplemented with unconjugated antibody to reach an antibody dose of 10, 30, or 100 μg. At 72 h post tracer injection, mice were sacrificed by cervical dislocation. Prior to that, mice received pimonidazole and Hoechst, as described above. Blood, muscle, tumor, lymph nodes, brown fat, thymus, heart, lungs, spleen, pancreas, liver, kidneys, stomach, duodenum, jejunum, ileum, colon, bone marrow, and bone were dissected, weighed, and activity was measured using a γ-counter (2480 Wizard, Perkin-Elmer). Results are presented as the percentage injected dose per gram tissue (%ID/g).

To determine the pharmacokinetics, mice with subcutaneous B16ova tumors received an i.v. injection via the tail vein of 3 μg of (0.33 MBq/μg) [^111^In]In-MSC3 or [^111^In]In-hIgG1, both supplemented to 30 μg of protein with unconjugated antibody. At 24, 48, or 72 h post tracer injection (*n* = 6), mice were sacrificed by cervical dislocation, and tumor and normal tissue uptake was determined as described previously.

### Quantitative CAIX MicroSPECT/CT Imaging

Mice with subcutaneous B16ova, B16F1, or MOC1.3D5 tumors (*n* = 6) received an i.v. injection in the tail vein of 10 μg (1.5 MBq/μg) [^111^In]In-MSC3 supplemented to 30 μg of protein with an unlabeled antibody. At 24 h post tracer injection, images of B16ova and MOC1.3D5 bearing mice were acquired with a U-SPECT-II/CT (MILabs) and of B16F1 bearing mice with a U-SPECT6/CT (MILabs). Scans were acquired under general anesthesia (isoflurane/O_2_) for 30 min using the mouse HS 1.0 mm pinhole collimator (U-SPECTII/CT) or the GP-M 0.60 mm pinhole collimator (U-SPECT6/CT), followed by a CT scan (U-SPECTII/CT: spatial resolution 160 μm, 65 kV, 0.615 mA or U-SPECT6/CT: spatial resolution 100 μm, 50 kV, 0.4 mA). Scans were reconstructed with MILabs reconstruction software, using an ordered-subset expectation maximization algorithm, with a voxel size of 0.2 mm^3^ and a Gaussian filter of 0.6 mm. Maximum intensity projections were created using VivoQuant. A three-dimensional (3D) volume of interest (VOI) was drawn around the tumor and CAIX^+^ area within the tumor using a fixed voxel intensity threshold (tumor area: USPECTII = 1.5 × 10^8^, USPECT6 = 2.7 × 10^3^ and CAIX^+^ area: USPECTII = 4.0 × 10^8^, U SPECT6 = 7.3 × 10^3^). The signal originating from liver or lung uptake in these regions of interest was identified using the corresponding CT image and was excluded. Using a standard series with known radioactivity, a calibration curve was determined for both scanners. *In vivo* tracer uptake within a VOI was quantified as the percentage injected dose per milliliter (%ID/mL).

### Autoradiography (AR) and Immunohistochemistry (IHC)

For AR analysis, frozen tumor sections (5 μm) from mice injected with [^111^In]In-MSC3 were mounted on poly-l-lysine coated slides and exposed to a Fujifilm BAS cassette 2025 (Fuji Photo Film). Cassettes were scanned using an AS-1800 II bioimaging analyzer at a pixel size of 25 × 25 μm. The same or consecutive sections were fixed in ice-cold acetone for 10 min, and IHC staining was performed to evaluate CAIX, pimonidazole, perfusion, and vessels, as described by Hoeben et al.^32^ For the IHC staining of CD45.2, sections were incubated (30 min, 37 °C) with biotinylated mouse anti-CD45.2 (Biolegend, 109804), diluted 1:100, followed by incubation with mouse anti-biotin Cy3 (Jackson Immunoresearch Laboratories), diluted 1:100 (45 min, 37 °C). Whole-tissue section grayscale images (pixel size, 2.59 × 2.59 μm) for vessels, perfusion, pimonidazole, CAIX, and CD45.2 were obtained and subsequently converted into binary images as previously described.^33^ Thresholds for segmentation of the fluorescent signals were manually set above the background signal for each individual marker. Binary images were used to calculate the CAIX fraction or pimonidazole relative to the total tumor area. The quantification of CD45.2 and CAIX fractions at different distances to perfused vasculature was done as previously described.^34^

### Spatial Correlation of Autoradiography and Immunohistochemistry

For the correlation of IHC images with AR images, AR images were inverted and overlaid using ImageJ (version 1.51s). The figure and pixel size of the IHC images were bicubically rescaled to match the AR images in iVision (version 4.5.5 r7). By parametric mapping, all viable tumor areas within IHC and AR grayscale images were divided into squares of 10 × 10 pixels.^33^ Gray values within these coregistered squares were compared between imaging modalities for spatial correlation analysis.

### Statistical Analyses

Statistical analyses were performed using GraphPad Prism (version 8.0). A paired *t*-test, unpaired *t*-test, or *t*-test with Welch correction, if SD was considered unequal, was used to compare groups. To compare >2 groups, one-way ANOVA or the Friedman test was used. The Spearman test was used to assess correlations between values. *P* values of 0.05 or less were considered significant. Results are expressed as mean value ± SD.

## Results

### [^111^In]In-MSC3 Specifically Binds to Hypoxic CAIX^+^ Tumor Cells *In Vitro*

**Figure 1:**
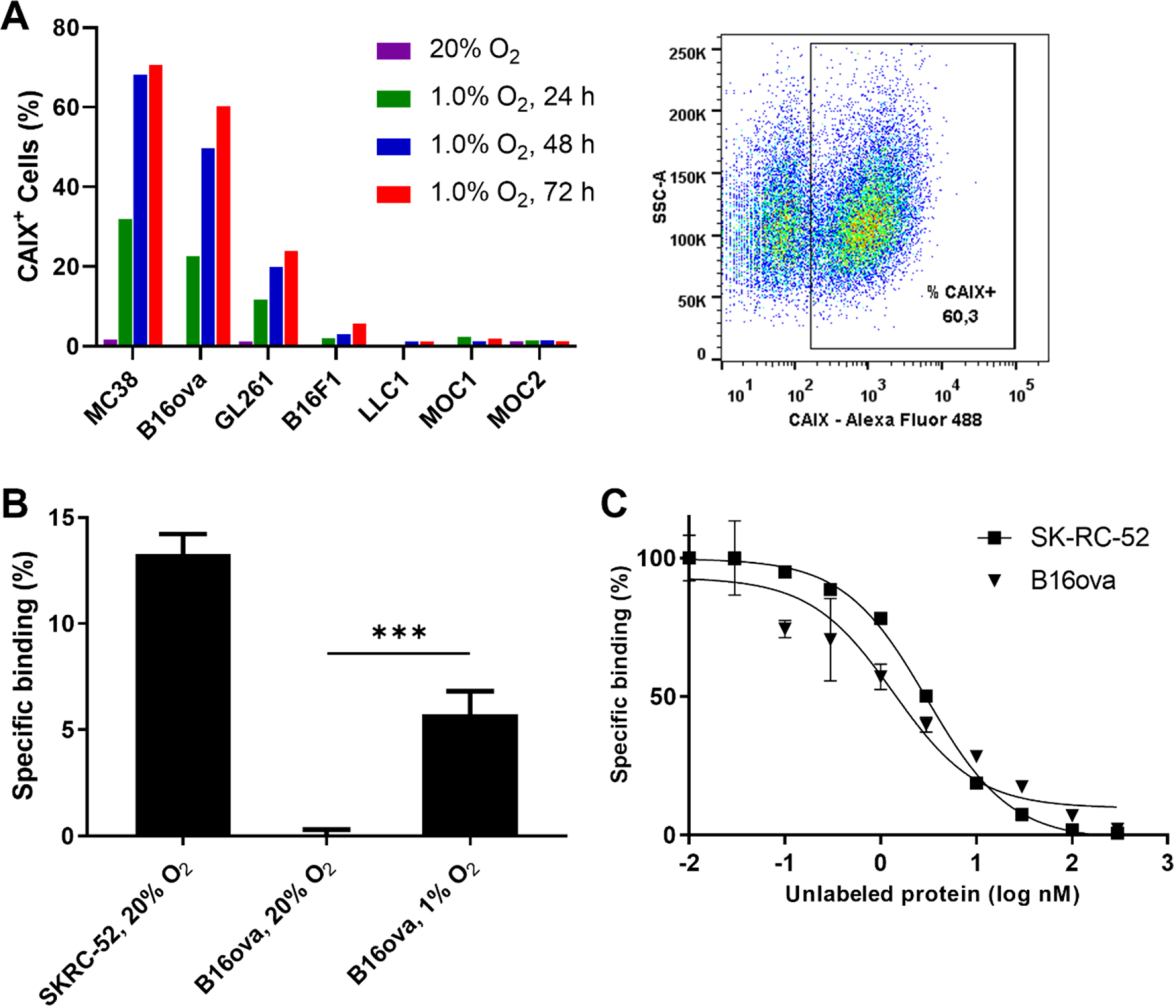
[^111^In]In-MSC3 bind specifically to CAIX^+^ tumor cells *in vitro*. (A) Percentage of CAIX^+^ tumor cells cultured at normoxic and hypoxic conditions for 24, 48, and 72 h by flow cytometry analysis for several mouse tumor cell lines. An example of gating of CAIX^+^ B16ova cells. (B) Specific binding of [^111^In]In-MSC3 to B16ova and SK-RC-52 cells. (C) IC_50_ analysis of [^111^In]In-MSC3 on B16ova cells (hypoxic conditions, 1% O_2_) and SK-RC-52 cells (normoxic conditions).

Flow cytometry showed inducibility of CAIX expression under hypoxic conditions in several murine tumor cell lines (Figure 1A). When cultured at normoxic (20% O_2_) conditions, none of the cell lines exhibit CAIX expression. When cultured at hypoxic conditions (1.0% O_2_), the number of CAIX^+^ cells increased in a time-dependent manner. MC38 and B16ova cells showed the highest inducibility of CAIX (71 and 60% at 72 h, respectively), while GL261 and B16F1 showed intermediate inducibility (24% and 5.7% at 72 h, respectively). In LLC1, MOC1, and MOC2 cells, almost no CAIX^+^ cells were detected regardless of the time of hypoxia.

To evaluate the binding specificity of [^111^In]In-MSC3 to murine and human CAIX, *in vitro* binding studies were performed using B16ova and control SK-RC-52 cells that constitutively overexpresses CAIX independent of the oxygen concentration.^35^ Under normoxic conditions, [^111^In]In-MSC3 showed specific binding to SK-RC-52 cells (13.3 ± 0.94%) (Figure 1B). In contrast, B16ova cells cultured under normoxic conditions showed no specific binding (0.02 ± 0.28%). However, when cultured for 72 h under hypoxic conditions, specific binding to B16ova cells was significantly increased (5.72 ± 1.09%, *P* = 0.0009). These data demonstrate that [^111^In]In-MSC3 binds specifically to hypoxic tumor cells. Furthermore, the IC_50_ of [^111^In]In-MSC3 for mCAIX was determined to be 1.36 ± 0.59 nM (B16ova cells, cultured under hypoxia), which did not differ significantly from that for hCAIX of 3.06 ± 0.24 nM (SK-RC-52 cells, cultured under normoxia) (Figure 1C).

### [^111^In]In-MSC3 Specifically Accumulates in Hypoxic CAIX^+^ Tumor Tissue *InVivo*

**Figure 2:**
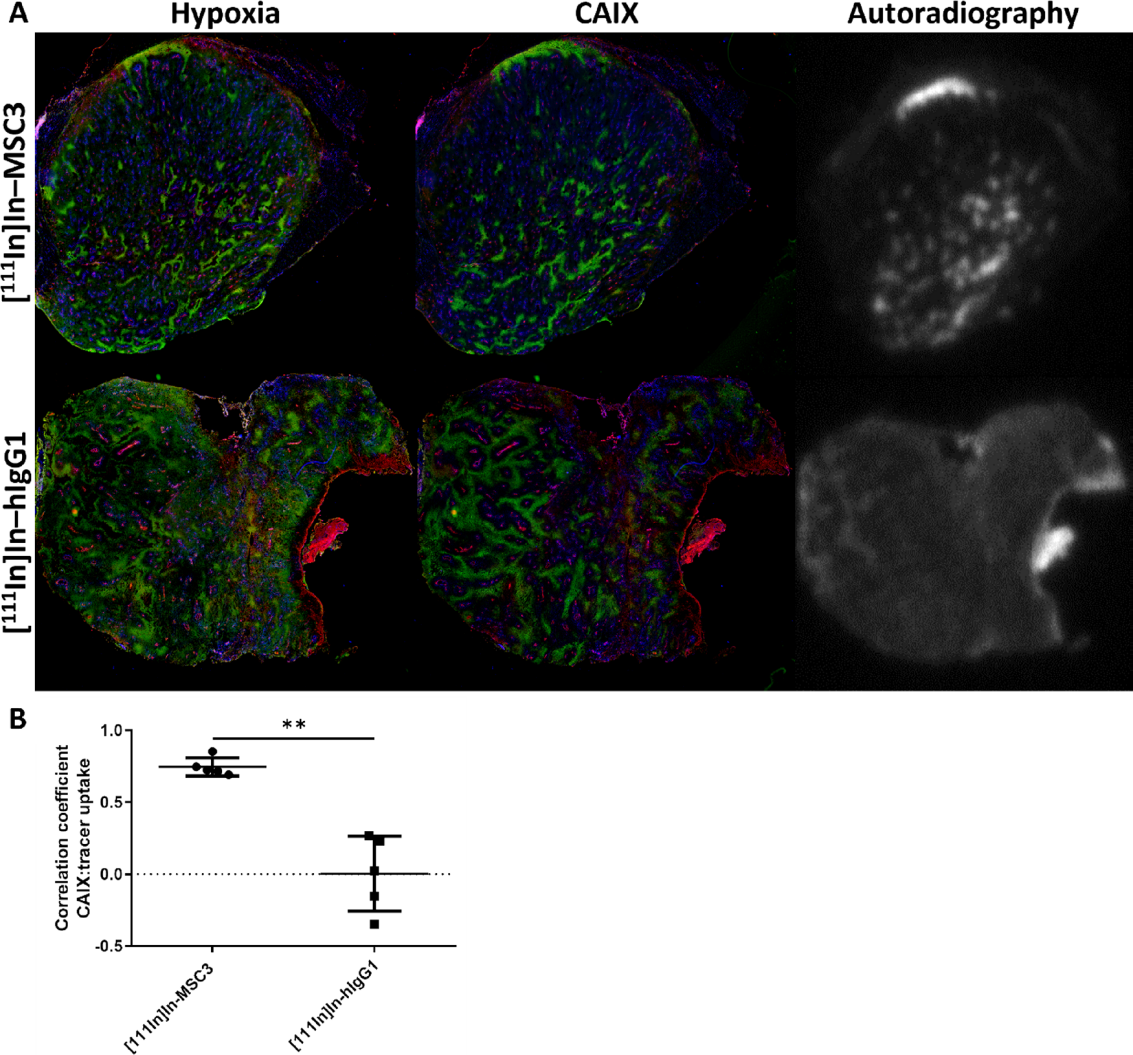
[^111^In]In-MSC3 specifically accumulates in CAIX^+^ tumor areas in B16ova tumors. (A) Immunohistochemical images and autoradiographic images of a B16ova tumor section injected with [^111^In]In-MSC3 or [^111^In]In-hIgG1. Vessels: red (9F1), perfusion: blue (Hoechst 33342), hypoxia: green (pimonidazole) (left column), and CAIX: green (middle column). (B) Spatial correlation between CAIX expression and tracer uptake as determined by coregistration and quantitative analysis of IHC and AR.

To verify the binding specificity of [^111^In]In-MSC3 to CAIX^+^ cells in hypoxic tumor areas *in vivo*, B16ova-tumor-bearing mice were injected with [^111^In]In-MSC3 or [^111^In]In-hIgG1. Tumor tissue was collected, and sections were analyzed by AR and IHC. [^111^In]In-MSC3 AR showed a clear colocalization of the radiotracer with the hypoxic cell marker (pimonidazole) and CAIX protein expression, while [^111^In]In-hIgG1 did not. (Figure 2A). Spearman correlation coefficients between AR and CAIX IHC were significantly higher (*P* = 0.002) for mice injected with [^111^In]In-MSC3 (*R*_s_ = 0.75 ± 0.06) compared with mice injected with [^111^In]In-hIgG1 (*R*_s_ = 0.00 ± 0.26) (Figure 2B).

### Antibody Protein Dose and Time Optimization of [^111^In]In-MSC3 in B16ova

**Figure 3:**
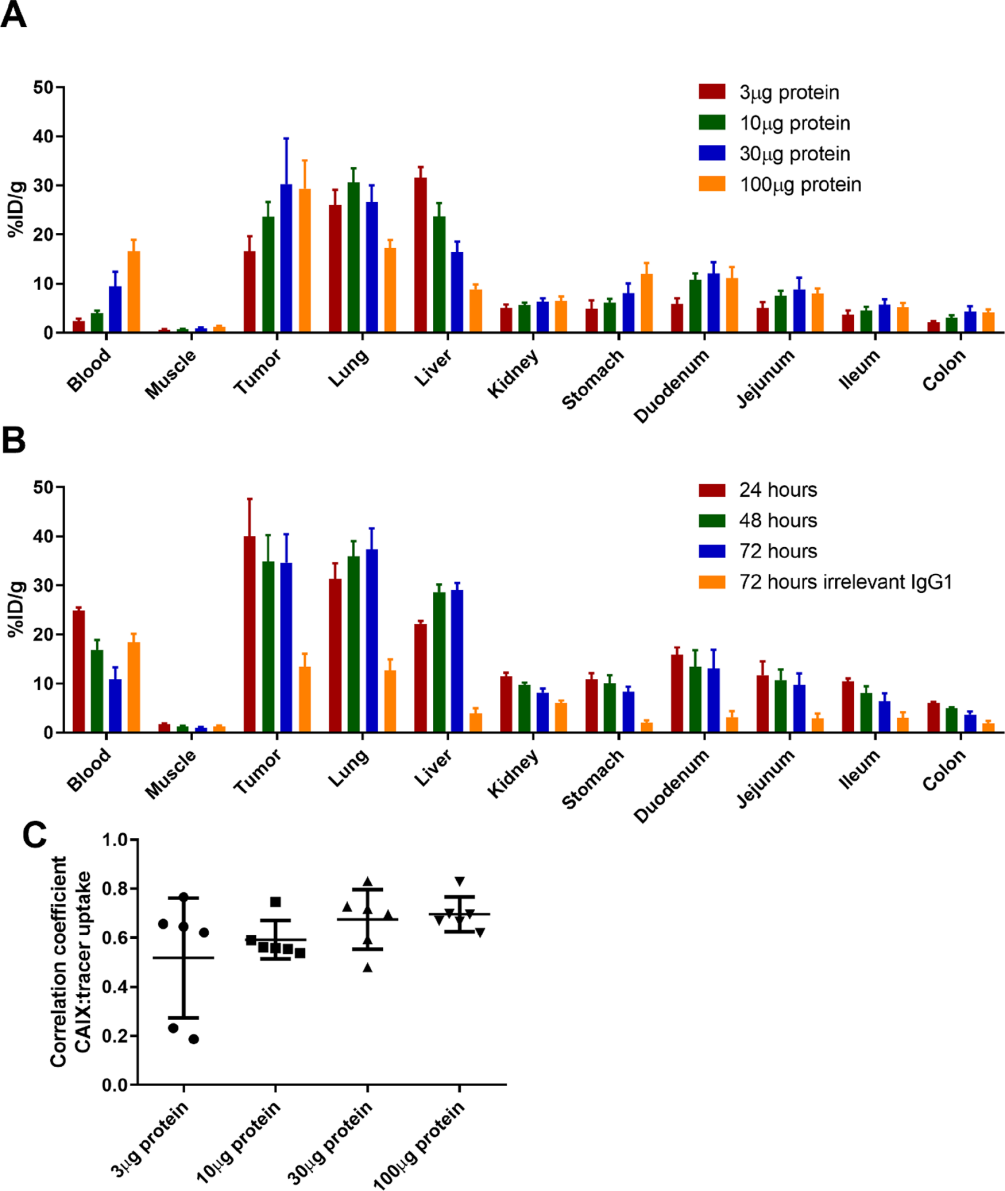
Dose and time-dependent biodistribution of [^111^In]In-MSC3 and [^111^In]In-hIgG1 in B16ova tumors and selected normal tissues. (A) Dose optimization for 3, 10, 30, and 100 of μg protein at 72 h post injection. (B) Time-dependent biodistribution at 24, 48, and 72 h post injection using 30 μg of protein. (C) Spatial correlation of CAIX expression with intratumoral tracer uptake as determined by the coregistration analysis of IHC and AR.

To evaluate *in vivo* distribution and optimize the protein dose of [^111^In]In-MSC3 to visualize the CAIX^+^ tissue, we performed a dose escalation study in mice bearing B16ova tumors. Tumor uptake of [^111^In]In-MSC3 was the highest at 30 μg of antibody (30.3 ± 9.4%ID/g) (Figure 3A). A protein dose of 3 μg resulted in a significantly lower tumor uptake compared to 30 μg (*P* = 0.004) and 100 μg (*P* = 0.006). A comparison between IHC and AR images revealed a stable correlation between radiotracer uptake and tumor CAIX expression for different doses, but an increased standard deviation for a dose of 3 μg was observed (*P* = 0.02) (Figure 3C). Significantly decreased radiotracer uptake was also observed in the liver at all dose levels compared to 3 μg protein (*P* < 0.0001) and for the lung at 100 μg (all *P* < 0.0003). Radiotracer concentration in the blood significantly increased at antibody doses 30 and 100 μg compared to 3 and 10 μg (all *P* < 0.0005), resulting in a decrease of the tumor-to-blood ratio (all *P* < 0.03) (Supporting Table S1). The tumor-to-muscle ratio remained high at all doses.

An antibody dose of 30 μg [^111^In]In-MSC3 was selected for a subsequent study to determine radiotracer retention in tumor and normal tissues. At 24 h post injection, tumor uptake of [^111^In]In-MSC3 was 40.0 ± 7.6%ID/g and remained stable up to 72 h post injection (Figure 3B and Supporting Table S2). Over time we observed clearance of the radiotracer from blood (all *P* < 0.005), resulting in a significantly increased tumor-to-blood ratio at 72 h compared to 24 h (*P* = 0.002) and 48 h (*P* = 0.02). Many other organs like brown fat, thymus, heart, pancreas, bone marrow, bone, colon, ileum, and kidneys showed decreased uptake at 72 h compared to 24 h post injection (all *P* < 0.009). Only uptake in the liver was increased at this time point (*P* < 0.0001). At 72 h post injection, [^111^In]In-MSC3 tumor uptake was significantly higher compared to uptake of irrelevant [^111^In]In-hIgG1 (*P* = 0.0002), while blood levels were lower (*P* = 0.0008). Liver, lung, spleen, pancreas, kidneys, stomach, duodenum, jejunum, ileum, and colon tissue showed lower uptake of [^111^In]In-hIgG1 compared with [111In]In-MSC3 (all *P* < 0.01). Full biodistribution data are described in Supporting Table S2.

### mCAIX MicroSPECT/CT Quantitatively Discriminates Tumor Models with High *vs* Low CAIX^+^ Fractions

**Figure 4:**
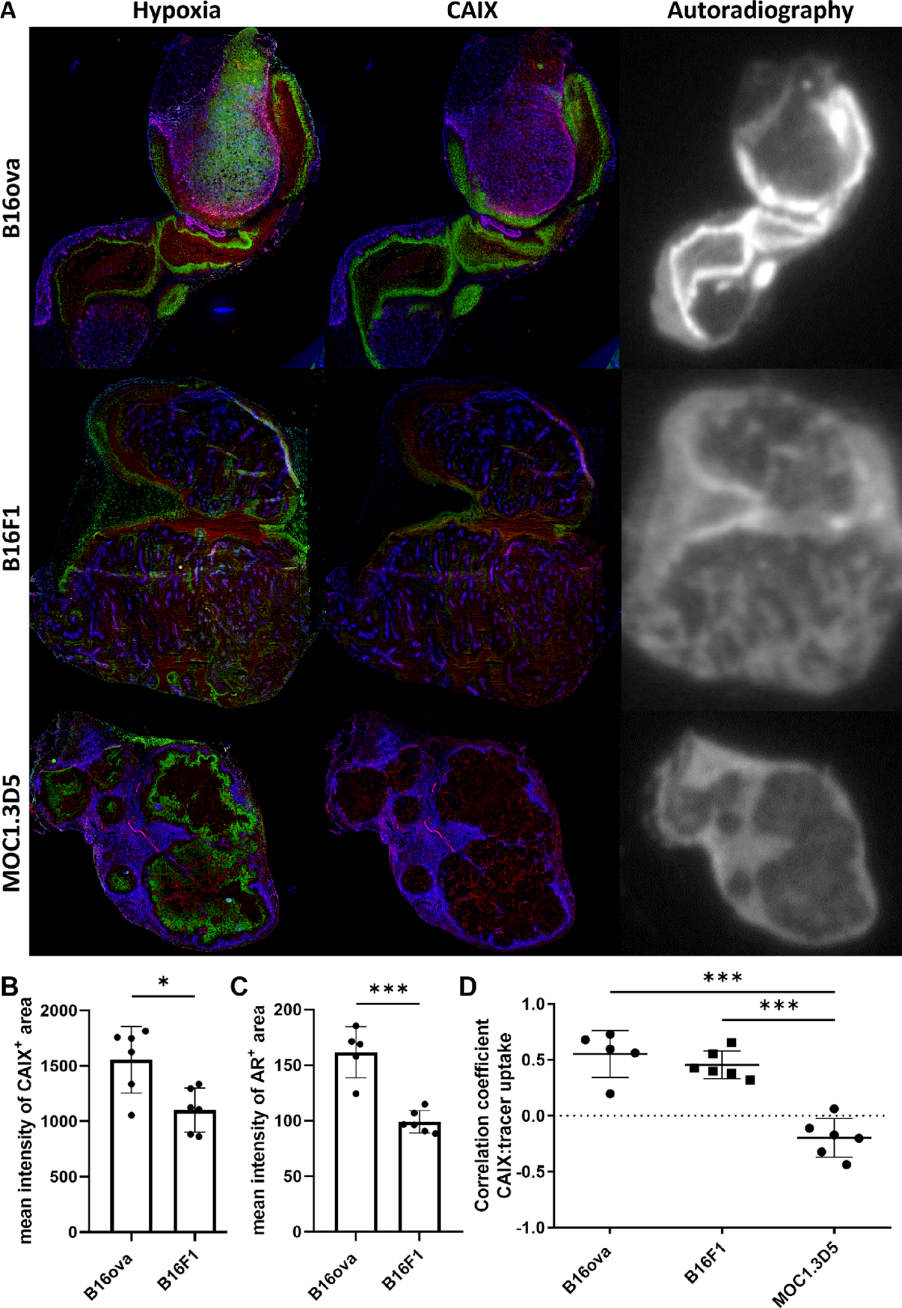
[^111^In]In-MSC3 specifically accumulates in CAIX^+^ tumors and can be used to discriminate tumor models with a larger CAIX^+^ fraction from tumor models with a smaller CAIX^+^ fraction by autoradiography. (A) Immunohistochemical images and autoradiographic images of a B16ova, MOC1.3D5, and B16F1 tumor sections of mice injected with [^111^In]In-MSC3. Vessels: red (9F1), perfusion: blue (Hoechst 33342), hypoxia: green (pimonidazole) (left column), and CAIX: green (middle column). (B) Mean gray value intensity per pixel of the CAIX signal in the CAIX^+^ area. (C) Mean gray value intensity per pixel of the AR signal in the AR^+^ area. (D) Spatial correlation of CAIX expression with intratumoral tracer uptake as determined by the coregistration analysis of the two modalities.

To evaluate the potential of [^111^In]In-MSC3 to discriminate tumors with a larger CAIX^+^ fraction from tumors with a smaller CAIX^+^ fraction, IHC, AR, and microSPECT/CT were performed in three different tumor models: B16ova, B16F1, and MOC1.3D5. IHC analysis revealed hypoxic tumor areas (pimonidazole) in B16ova, MOC1.3D5, and B16F1 tumors (Figure 4A). These hypoxic areas colocalized strongly with CAIX expression in B16ova and mildly in B16F1 but not in MOC1.3D5 tumors, which lack CAIX expression. Quantitative IHC demonstrated a comparable fraction of CAIX^+^ area in B16ova and B16F1 (Supporting Figure S1) but a significantly higher intensity of the CAIX signal in B16ova compared to B16F1 (*P* = 0.01) (Figure 4B).

AR images showed high [^111^In]In-MSC3 uptake in the tumor tissue of B16ova, moderate uptake in B16F1 tumors, and the lowest uptake in MOC1.3D5 tumors. For MOC1.3D5, tracer uptake was observed in the well-perfused rim of MOC1.3D5 tumors, but this did not correlate with CAIX expression and was thus regarded as nonspecific uptake (Figure 4A). Quantification of the tracer uptake on AR demonstrated a significantly higher intensity in AR^+^ areas of B16ova compared to B16F1 tumors (*P* = 0.0002) (Figure 4C), which is in line with the IHC findings. A clear spatial correlation of CAIX expression and tracer uptake was observed for B16ova and B16F1 but not for MOC1.3D5 (Figure 4D).

**Figure 5:**
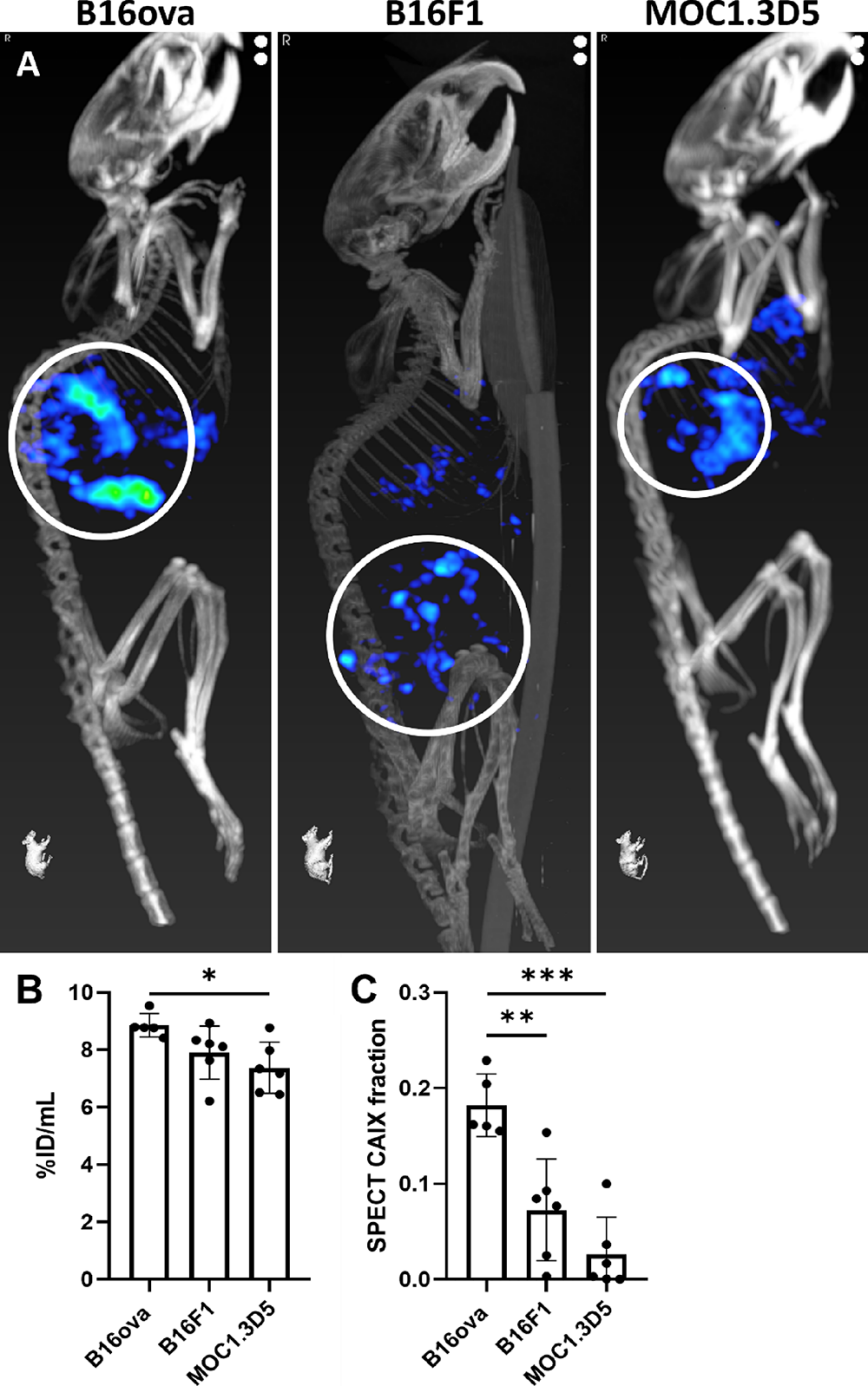
[^111^In]In-MSC3 specifically accumulates in CAIX^+^ tumors and can be used to discriminate tumor models with a larger CAIX^+^ fraction from tumor models with a smaller CAIX^+^ fraction by microSPECT/CT. (A) Maximum intensity projections of mCAIX microSPECT/CT images of three tumor models. Tumors are indicated by white circles. (B) *In vivo* tracer uptake (%ID/mL) in total tumor volume. (C) CAIX^+^ fraction of tumors determined by microSPECT quantification.

Representative examples of mCAIX microSPECT/CT scans for the three tumor models are presented in Figure 5A. The intensity of the CAIX signal in the scans was quantified as the %ID/mL in the whole tumor volume (Figure 5B), which was significantly higher for B16ova compared to MOC1.3D5 tumors (*P* = 0.02) but not compared to B16F1 tumors. By determining the CAIX^+^ tumor volume and the total tumor volume, the CAIX^+^ fraction per tumor was calculated (Figure 5C). B16ova tumors showed a significantly higher CAIX^+^ fraction compared to B16F1 (*P* = 0.002) and MOC1.3D5 tumors (*P* < 0.0001). Intratumoral heterogeneity of radiotracer uptake was visualized and related to a plane in the vicinity of the section used for AR (Supporting Figure S2). These data illustrate the sensitivity of mCAIX microSPECT/CT and AR to detect tumors with varying CAIX expression.

### CAIX^+^ Areas are Less Infiltrated by Leukocytes in B16ova Tumors

**Figure 6:**
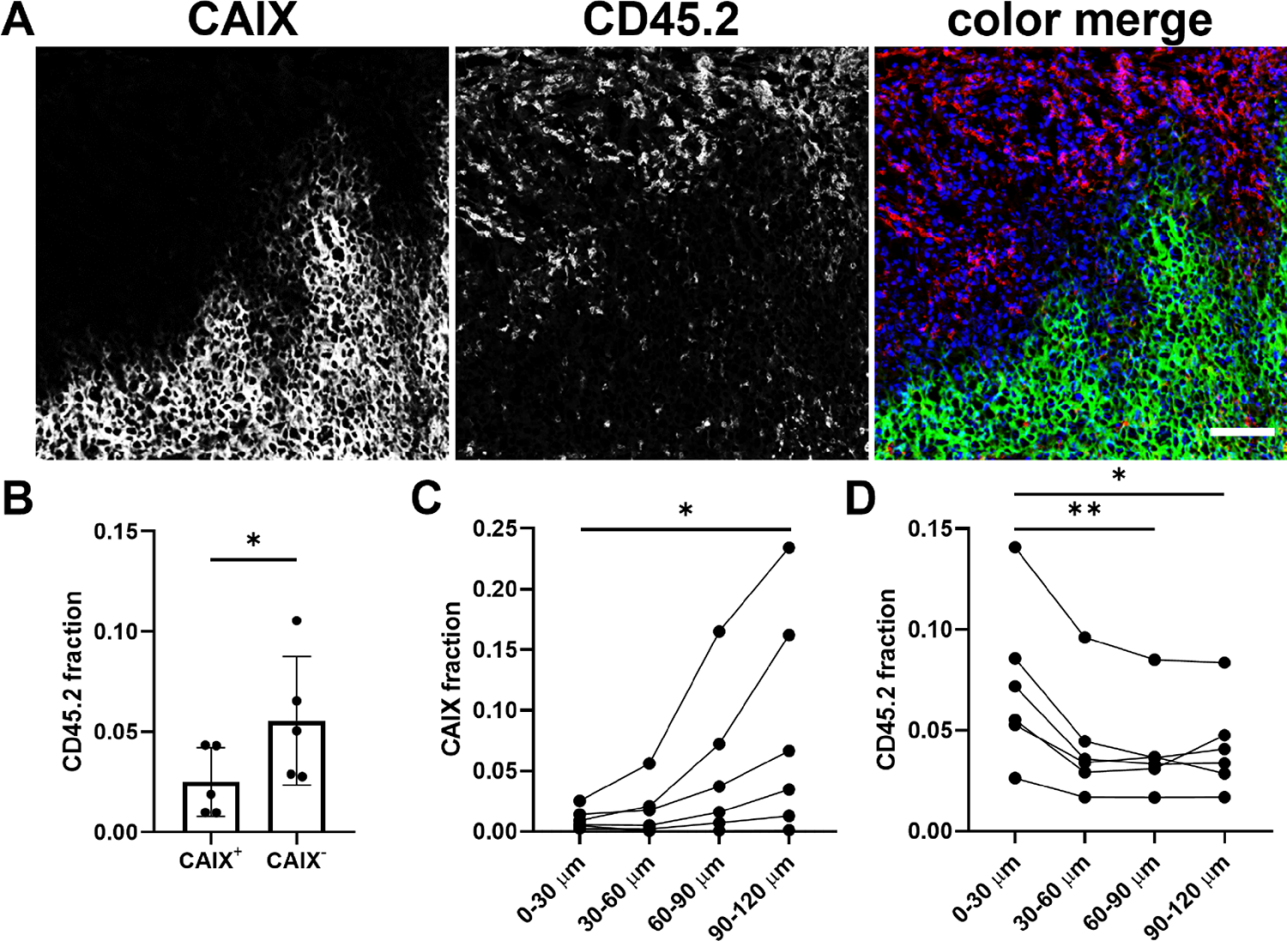
CD45.2^+^ leukocytes are less present in CAIX^+^ areas in B16ova tumors at a greater distance to vessels. (A) Gray value images of the selected viable tumor tissue of a B16ova tumor showing CAIX, leukocytes (CD45.2) and a color merge of CAIX: green, leukocytes: red, and nuclei: blue (Hoechst 33342). Magnification 20×. The scale bar represents 100 μm. (B) CD45.2 fraction in CAIX^+^ and CAIX^–^ areas of the elected viable tumor tissue, *n* = 5. Four regions (636 × 636 μm^2^) per mouse. (C) CAIX fraction at variable distances to perfused vessels, *n* = 6. (D) CD45.2 fraction at variable distances to perfused vessels, *n* = 6.

We next quantified the number of leukocytes present in CAIX^–^ and CAIX^+^ areas of B16ova tumors. Infiltration of CD45.2^+^ leukocytes in these tumor areas was assessed on tumor sections in four representative regions (636 × 636 μm) of viable tumor tissue. Gray value images showed a disparity between the localization of the CAIX signal and the CD45.2 signal that was also observed in a color merge of both images (Figure 6A). When quantified, the CD45.2 fraction in CAIX^+^ areas was significantly lower compared to that in CAIX^–^ areas (*P* = 0.03) (Figure 6B). Analysis of MOC1.3D5 tumors, which lack CAIX expression but are hypoxic, showed a similar disparity between CD45.2 infiltration and pimonidazole localization (*P* = 0.02) (Supporting Figure S3), underlining the diminished immune cell presence in hypoxic tumor areas. Further analysis revealed an inverse correlation between CAIX expression and CD45.2 localization relative to the vasculature. The CAIX fraction increased at a greater distance to perfused vessels, while the CD45.2 fraction decreased further away from the vasculature (Figure 6C–D), indicative of chronic, diffusion-limited hypoxia being associated with CAIX positivity and immune cell limitation. CAIX and CD45.2 fractions did not correlate to tumor size.

## Discussion

Tumor hypoxia plays an important role in resistance to radio- and immunotherapy. To overcome this, new therapeutic approaches are being developed aiming at reducing tumor hypoxia. However, in clinical trials using hypoxia modification and targeted therapies, hypoxia assessment is rarely used for patient stratification. Due to this lack of patient selection, a therapeutic effect for the subset of the patient population with hypoxic tumors is potentially overlooked.^36^ In light of this, accurate assessment of hypoxia in clinical and preclinical studies is essential.^10,11,37,38^ However, as also shown in our study, hypoxia and the hypoxic biomarker CAIX are distributed heterogeneously throughout the tumor. Therefore, tumor biopsies or sections may not be representative of the whole tumor lesion.^39^ Moreover, immunohistochemistry of tumor sections does not allow longitudinal monitoring of the hypoxia biomarker CAIX. Here, we develop mCAIX microSPECT/CT and demonstrate its potential to evaluate chronic hypoxia in syngeneic mouse tumors and show that the presence of CAIX correlates with hypoxia and low infiltration of immune cells.

*In vitro*, we show that the inducibility of CAIX is highly variable between different murine tumor cell lines. Although a strong correlation exists between CAIX expression and hypoxia, discrepancies can also be found in human tumors.^40−42^ This disparity makes CAIX imaging unsuitable for hypoxia assessment in some tumor models. For instance, MC38 colon carcinoma cells show the highest *in vitro* CAIX inducibility, but *in vivo* CAIX expression is very low.^43^ The absence of CAIX in MC38 tumor tissue suggests that hypoxia kinetics are important for the induction of CAIX. The time MC38 cells are hypoxic before they become necrotic might, for instance, be too brief to accumulate a significant amount of CAIX.

To this end, we selected B16ova for the in vitro and in vivo characterization of [^111^In]In-MSC3. Our *in vivo* studies show accumulation of [^111^In]In-MSC3, specifically in CAIX^+^ areas, which largely colocalizes with hypoxia (determined by pimonidazole). If any discrepancy in CAIX expression and tracer uptake is observed, this can be largely attributed to nonspecific uptake in highly perfused areas. This is especially the case for the negative correlation in mice injected with isotype control antibody [^111^In]In-hIgG1.

High tumor uptake is observed for antibody doses of 10–100 μg [^111^In]In-MSC3. By escalating antibody dose, tracer uptake in the liver and lungs can be saturated, resulting in higher antibody concentrations in the blood and as a consequence, enhanced tumor uptake. Significantly higher uptake of [^111^In]In-MSC3 compared to irrelevant [^111^In]In-hIgG1 in several organs suggests CAIX-mediated uptake. CAIX expression in the mouse gastrointestinal tract has been described as well as mild expression in the lungs, kidneys, pancreas, liver, and spleen.^44,45^ Over time, [^111^In]In-MSC3 clears from the blood and other tissues, resulting in a lower background signal. Despite this, we decided to image at 24 h post tracer injection because of the growth characteristics of our model systems. Murine tumor models like B16ova and B16F1 have a doubling time of approx. 2 days.^28,46^ Therefore, imaging at an early time point would introduce fewer changes in spatial tumor characteristics between tracer injection and imaging. Imaging modalities using rapidly clearing radiotracers such as affibody molecules or antibody fragments allow for imaging at even earlier time points post injection. However, as we have shown previously, the rapid clearance of these tracers likely exceeds the time that is necessary for sufficient tracer accumulation in hypoxic areas, thereby hampering its potential for CAIX imaging in this setting.^47^

[^111^In]In-MSC3 discriminates between tumors with high versus low CAIX expression. For example, radiotracer uptake was higher in CAIX^+^ tumor areas compared with CAIX^–^ tumor areas (*ex vivo* AR). Furthermore, CAIX^+^ tumors show a higher CAIX^+^ fraction compared with CAIX^–^ tumors (*in vivo* microSPECT/CT). The total tumor uptake (%ID/mL) shows only small differences between CAIX^+^ and CAIX^–^ tumor models. This is probably due to the relatively small fraction of the tumor where the tracer specifically binds compared to the nonhypoxic tumor volume. CAIX^–^ MOC1.3D5 tumors sometimes show radiotracer uptake in the tumor rim, which is, based on the IHC analyses, most likely attributed to uptake in the well-perfused stroma and thus considered as nonspecific binding.

In recent years, it has become clear that the characteristics of the tumor microenvironment, including tumor hypoxia, contribute to tumor progression and therapy resistance.^36^ Hypoxic areas are also known to have a decreased infiltration of immune cells.^16^ In B16ova tumors, we demonstrate that CAIX^+^ tumor areas contain a lower number of infiltrating leukocytes compared with CAIX^–^ tumor areas. In MOC1.3D5 tumors, which are hypoxic but do not show CAIX expression, a similar effect is observed. Moreover, in relation to distance to the vessels, CAIX expression increases with an increased distance from the perfused vasculature, which is in line with the hypoxia marker pimonidazole.^34^ In contrast, leukocyte presence is more concentrated in close proximity to the vessels. However, further analysis of specific immune cell subsets, like NK and T cells, in syngeneic mouse models is needed to fully understand the consequences of hypoxia on antitumor immunity. Recently, others have described such relations between CAIX and immune cell infiltration. In head and neck cancer, an inverse correlation between CAIX expression and CD3^+^ tumor-infiltrating lymphocytes (TIL) was reported.^48^ Additionally, head and neck patients with a CAIX_high_/CD3^+^ TIL_low_ phenotype do worse in terms of locoregional control.^49^ In colorectal cancer, a similar correlation was observed, where CD3, CD4, and CD8 immune cold phenotypes were associated with increased hypoxia and CAIX expression and were prognostic for poor overall survival.^50^ The upregulation of PD-L1 and other immune checkpoints was associated with increased CAIX expression in lung and breast cancer.^51,52^ Interestingly, response to PD-1 blockade therapy was associated with a CAIX_low_/CD8_high_ phenotype in melanoma and head and neck squamous cell carcinoma patients, suggesting that CAIX might be one of the predictive markers for immunotherapy response.^13,14^

## Conclusions

Together, our results demonstrate that mCAIX microSPECT/CT can successfully detect heterogeneous CAIX-expressing areas in syngeneic murine tumor models that, compared to normoxic tumor areas, show less infiltration by immune cells. In the future, this imaging modality holds the potential for noninvasive response monitoring of hypoxia-targeted therapies in immunocompetent mouse models, which is especially important in the context of immunotherapy research.

## Supporting Information Available

Fraction CAIX^+^ area in several tumor models analyzed by IHC (Figure S1); cross sections of microSPECT and AR images matched approximately (Figure S2); CD45.2 fraction in hypoxic (pimonidazole^+^) and normoxic (pimonidazole^–^) tumor areas of MOC1.3D5 whole tumor sections, *n* = 6 (Figure S3); tumor-to-background ratio for dose and time optimization in mice bearing B16ova tumors (Table S1); and dose and time optimization biodistribution data of [^111^In]In-mCAIX and [111In]In-hIgG1 in B16ova tumors and selected normal tissues (Table S2) (PDF)

## List of Abbreviations

AR: autoradiography
CAIX: carbonic anhydrase IX
CD3/4/8/45.2: cluster of differentiation
CT: computed tomography
DTPA: diethylenetriaminepentaacetic acid
FCS: fetal calf serum
IHC: immunohistochemistry
^111^In: indium-111
I.V.: intravenous
PBS: phosphate-buffered saline
SPECT: single-photon emission computerized tomography
TIL: tumor-infiltrating lymphocyte
VOI: volume of interest

## References

[ref1] Span P. N.; Bussink J. Biology of hypoxia. Semin. Nucl. Med. 2015, 45, 101–109. 10.1053/j.semnuclmed.2014.10.002. 25704383

[ref2] Vito A.; El-Sayes N.; Mossman K. Hypoxia-Driven Immune Escape in the Tumor Microenvironment. Cells 2020, 9, 992 10.3390/cells9040992. 32316260 PMC7227025

[ref3] Scharping N. E.; Rivadeneira D. B.; Menk A. V.; Vignali P. D. A.; Ford B. R.; Rittenhouse N. L.; et al. Mitochondrial stress induced by continuous stimulation under hypoxia rapidly drives T cell exhaustion. Nat. Immunol. 2021, 22, 205–215. 10.1038/s41590-020-00834-9. 33398183 PMC7971090

[ref4] Vaupel P.; Kallinowski F.; Okunieff P. Blood flow, oxygen and nutrient supply, and metabolic microenvironment of human tumors: a review. Cancer Res. 1989, 49, 6449–6465. 2684393

[ref5] Secomb T. W.; Hsu R.; Ong E. T.; Gross J. F.; Dewhirst M. W. Analysis of the effects of oxygen supply and demand on hypoxic fraction in tumors. Acta Oncol. 1995, 34, 313–316. 10.3109/02841869509093981. 7779415

[ref6] Taylor C. T.; Colgan S. P. Regulation of immunity and inflammation by hypoxia in immunological niches. Nat. Rev. Immunol. 2017, 17, 774–785. 10.1038/nri.2017.103. 28972206 PMC5799081

[ref7] Nordsmark M.; Bentzen S. M.; Rudat V.; Brizel D.; Lartigau E.; Stadler P.; et al. Prognostic value of tumor oxygenation in 397 head and neck tumors after primary radiation therapy. An international multi-center study. Radiother. Oncol. 2005, 77, 18–24. 10.1016/j.radonc.2005.06.038. 16098619

[ref8] Toustrup K.; Sørensen B. S.; Lassen P.; Wiuf C.; Alsner J.; Overgaard J. Gene expression classifier predicts for hypoxic modification of radiotherapy with nimorazole in squamous cell carcinomas of the head and neck. Radiother. Oncol. 2012, 102, 122–129. 10.1016/j.radonc.2011.09.010. 21996521

[ref9] Schütze C.; Bergmann R.; Brüchne K.; Mosch B.; Yaromina A.; Zips D.; et al. Effect of [(18)F]FMISO stratified dose-escalation on local control in FaDu hSCC in nude mice. Radiother. Oncol. 2014, 111, 81–87. 10.1016/j.radonc.2014.02.005. 24636842

[ref10] Zannella V. E.; Dal Pra A.; Muaddi H.; McKee T. D.; Stapleton S.; Sykes J.; et al. Reprogramming metabolism with metformin improves tumor oxygenation and radiotherapy response. Clin. Cancer Res. 2013, 19, 6741–6750. 10.1158/1078-0432.CCR-13-1787. 24141625

[ref11] Skwarski M.; McGowan D. R.; Belcher E.; Di Chiara F.; Stavroulias D.; McCole M.; et al. Mitochondrial Inhibitor Atovaquone Increases Tumor Oxygenation and Inhibits Hypoxic Gene Expression in Patients with Non-Small Cell Lung Cancer. Clin. Cancer Res. 2021, 27, 2459–2469. 10.1158/1078-0432.CCR-20-4128. 33597271 PMC7611473

[ref12] Scharping N. E.; Menk A. V.; Whetstone R. D.; Zeng X.; Delgoffe G. M. Efficacy of PD-1 Blockade Is Potentiated by Metformin-Induced Reduction of Tumor Hypoxia. Cancer Immunol. Res. 2017, 5, 9–16. 10.1158/2326-6066.CIR-16-0103. 27941003 PMC5340074

[ref13] Zandberg D. P.; Menk A. V.; Velez M.; Normolle D.; DePeaux K.; Liu A.; et al. Tumor hypoxia is associated with resistance to PD-1 blockade in squamous cell carcinoma of the head and neck. J. ImmunoTher. Cancer 2021, 9, e002088 10.1136/jitc-2020-002088. 33986123 PMC8126285

[ref14] Najjar Y. G.; Menk A. V.; Sander C.; Rao U.; Karunamurthy A.; Bhatia R.; et al. Tumor cell oxidative metabolism as a barrier to PD-1 blockade immunotherapy in melanoma. JCI Insight 2019, 4, e124989 10.1172/jci.insight.124989. 30721155 PMC6483505

[ref15] Gaipl U. S.; Multhoff G.; Scheithauer H.; Lauber K.; Hehlgans S.; Frey B.; Rödel F. Kill and spread the word: stimulation of antitumor immune responses in the context of radiotherapy. Immunotherapy 2014, 6, 597–610. 10.2217/imt.14.38. 24896628

[ref16] Boreel D. F.; Span P. N.; Heskamp S.; Adema G. J.; Bussink J. Targeting Oxidative Phosphorylation to Increase the Efficacy of Radio- and Immune-Combination Therapy. Clin. Cancer Res. 2021, 27, 2970–2978. 10.1158/1078-0432.CCR-20-3913. 33419779

[ref17] Spigel D. R.; Faivre-Finn C.; Gray J. E.; Vicente D.; Planchard D.; Paz-Ares L.; et al. Five-Year Survival Outcomes From the PACIFIC Trial: Durvalumab After Chemoradiotherapy in Stage III Non-Small-Cell Lung Cancer. J. Clin. Oncol. 2022, 40, 1301–1311. 10.1200/JCO.21.01308. 35108059 PMC9015199

[ref18] Jiang B. H.; Semenza G. L.; Bauer C.; Marti H. H. Hypoxia-inducible factor 1 levels vary exponentially over a physiological relevant range of O2 tension. Am. J. Physiol.-Cell Physiol. 1996, 271, c1172–c80. 10.1152/ajpcell.1996.271.4.C1172. 8897823

[ref19] Meijer T. W.; Kaanders J. H.; Span P. N.; Bussink J. Targeting hypoxia, HIF-1, and tumor glucose metabolism to improve radiotherapy efficacy. Clin. Cancer Res. 2012, 18, 5585–5594. 10.1158/1078-0432.CCR-12-0858. 23071360

[ref20] Wykoff C. C.; Beasley N. J. P.; Watson P. H.; Turner K. J.; Pastorek J.; Sibtain A.; et al. Hypoxia-inducible expression of tumor-associated carbonic anhydrases. Cancer Res. 2000, 60, 7075–7083. 11156414

[ref21] Forker L.; Gaunt P.; Sioletic S.; Shenjere P.; Potter R.; Roberts D.; et al. The hypoxia marker CAIX is prognostic in the UK phase III VorteX-Biobank cohort: an important resource for translational research in soft tissue sarcoma. Br. J. Cancer 2018, 118, 698–704. 10.1038/bjc.2017.430. 29235571 PMC5846059

[ref22] Ong C. H. C.; Lee D. Y.; Lee B.; Li H.; Lim J. C. T.; Lim J. X.; et al. Hypoxia-regulated carbonic anhydrase IX (CAIX) protein is an independent prognostic indicator in triple negative breast cancer. Breast Cancer Res. 2022, 24, 38 10.1186/s13058-022-01532-0. 35659359 PMC9164406

[ref23] Bussink J.; van Herpen C. M. L.; Kaanders J. H. A. M.; Oyen W. J. G. PET-CT for response assessment and treatment adaptation in head and neck cancer. Lancet Oncol. 2010, 11, 661–669. 10.1016/S1470-2045(09)70353-5. 20226735

[ref24] Shin K. H.; Diaz-Gonzalez J. A.; Russell J.; Chen Q.; Burgman P.; Li X. F.; Ling C. C. Detecting changes in tumor hypoxia with carbonic anhydrase IX and pimonidazole. Cancer Biol. Ther. 2007, 6, 70–75. 10.4161/cbt.6.1.3550. 17172824

[ref25] Rafajová M.; Zatovicová M.; Kettman R.; Pastorek J.; Pastoreková S. Induction by hypoxia combined with low glucose or low bicarbonate and high posttranslational stability upon reoxygenation contribute to carbonic anhydrase IX expression in cancer cells. Int. J. Oncol. 2004, 24, 995–1004. 10.3892/ijo.24.4.995. 15010840

[ref26] Vordermark D.; Kaffer A.; Riedl S.; Katzer A.; Flentje M. Characterization of carbonic anhydrase IX (CA IX) as an endogenous marker of chronic hypoxia in live human tumor cells. Int. J. Radiat. Oncol., Biol., Phys. 2005, 61, 1197–1207. 10.1016/j.ijrobp.2004.11.031. 15752902

[ref27] Huizing F. J.; Hoeben B. A. W.; Franssen G. M.; Boerman O. C.; Heskamp S.; Bussink J. Quantitative Imaging of the Hypoxia-Related Marker CAIX in Head and Neck Squamous Cell Carcinoma Xenograft Models. Mol. Pharm. 2019, 16, 701–708. 10.1021/acs.molpharmaceut.8b00950. 30550290 PMC6364270

[ref28] Falo L. D.; Kovacsovics-Bankowski M.; Thompson K.; Rock K. L. Targeting antigen into the phagocytic pathway in vivo induces protective tumour immunity. Nat. Med. 1995, 1, 649–653. 10.1038/nm0795-649. 7585145

[ref29] Peske J. D.; Thompson E. D.; Gemta L.; Baylis R. A.; Fu Y. X.; Engelhard V. H. Effector lymphocyte-induced lymph node-like vasculature enables naive T-cell entry into tumours and enhanced anti-tumour immunity. Nat. Commun. 2015, 6, 7114 10.1038/ncomms8114. 25968334 PMC4435831

[ref30] Leick K. M.; Pinczewski J.; Mauldin I. S.; Young S. J.; Deacon D. H.; Woods A. N.; et al. Patterns of immune-cell infiltration in murine models of melanoma: roles of antigen and tissue site in creating inflamed tumors. Cancer Immunol. Immunother. 2019, 68, 1121–1132. 10.1007/s00262-019-02345-5. 31134297 PMC6887106

[ref31] Heskamp S.; Hobo W.; Molkenboer-Kuenen J. D.; Olive D.; Oyen W. J.; Dolstra H.; Boerman O. C. Noninvasive Imaging of Tumor PD-L1 Expression Using Radiolabeled Anti-PD-L1 Antibodies. Cancer Res. 2015, 75, 2928–2936. 10.1158/0008-5472.CAN-14-3477. 25977331

[ref32] Hoeben B. A.; Kaanders J. H.; Franssen G. M.; Troost E. G.; Rijken P. F.; Oosterwijk E.; et al. PET of hypoxia with 89Zr-labeled cG250-F(ab’)2 in head and neck tumors. J. Nucl. Med. 2010, 51, 1076–1083. 10.2967/jnumed.109.073189. 20554724

[ref33] Rademakers S. E.; Rijken P. F.; Peeters W. J.; Nijkamp M. M.; Barber P. R.; van der Laak J.; et al. Parametric mapping of immunohistochemically stained tissue sections; a method to quantify the colocalization of tumor markers. Cell Oncol. 2011, 34, 119–129. 10.1007/s13402-010-0008-2. PMC3063563 21302028

[ref34] Rijken P. F.; Bernsen H. J. J. A.; Peters J. P. W.; Hodgkiss R. J.; Raleigh J. A.; van de Kogel A. J. Spatial relationship between hypoxia and the (perfused) vascular network in a human glioma xenograft: a quantitative multi-parameter analysis. Int. J. Radiat. Oncol., Biol., Phys. 2000, 48, 571–582. 10.1016/S0360-3016(00)00686-6. 10974478

[ref35] Krieg M.; Haas R.; Brauch H.; Acker T.; Flamme I.; Plate K. D. Up-regulation of hypoxia-inducible factors HIF-1a and HIF-2a under normoxic conditions in renal carcinoma cells by von Hippel-Lindau tumor suppressor gene loss of function. Oncogene 2000, 19, 5435–5443. 10.1038/sj.onc.1203938. 11114720

[ref36] Walsh J. C.; Lebedev A.; Aten E.; Madsen K.; Marciano L.; Kolb H. C. The clinical importance of assessing tumor hypoxia: relationship of tumor hypoxia to prognosis and therapeutic opportunities. Antioxid. Redox Signaling 2014, 21, 1516–1554. 10.1089/ars.2013.5378. PMC4159937 24512032

[ref37] Han K.; Fyles A.; Shek T.; Croke J.; Dhani N.; D’Souza D.; et al. A Phase II Randomized Trial of Chemoradiation with or without Metformin in Locally Advanced Cervical Cancer. Clin. Cancer Res. 2022, 28, 5263–5271. 10.1158/1078-0432.CCR-22-1665. 36037303

[ref38] De Bruycker S.; Vangestel C.; Van den Wyngaert T.; Pauwels P.; Wyffels L.; Staelens S. (18)F-Flortanidazole Hypoxia PET Holds Promise as a Prognostic and Predictive Imaging Biomarker in a Lung Cancer Xenograft Model Treated with Metformin and Radiotherapy. J. Nucl. Med. 2019, 60, 34–40. 10.2967/jnumed.118.212225. 29980581

[ref39] Lukovic J.; Han K.; Pintilie M.; Chaudary N.; Hill R. P.; Fyles A.; Milosevic M. Intratumoral heterogeneity and hypoxia gene expression signatures: Is a single biopsy adequate?. Clin. Transl. Radiat. Oncol. 2019, 19, 110–115. 10.1016/j.ctro.2019.09.006. 31650046 PMC6804682

[ref40] Rademakers S. E.; Lok J.; van de Kogel A. J.; Bussink J.; Kaanders J. H. Metabolic markers in relation to hypoxia; staining patterns and colocalization of pimonidazole, HIF1a, CAIX, LDH-5, GLUT-1, MCT1 and MCT4. BMC Cancer 2011, 11, 167 10.1186/1471-2407-11-167. 21569415 PMC3115911

[ref41] Rademakers S. E.; Hoogsteen I. J.; Rijken P. F.; Oosterwijk E.; Terhaard C. H.; Doornaert P. A.; et al. Pattern of CAIX expression is prognostic for outcome and predicts response to ARCON in patients with laryngeal cancer treated in a phase III randomized trial. Radiother. Oncol. 2013, 108, 517–522. 10.1016/j.radonc.2013.04.022. 23719582

[ref42] Troost E. G.; Bussink J.; Kaanders J. H.; van Eerd J.; Peters J. P.; Rijken P. F.; et al. Comparison of different methods of CAIX quantification in relation to hypoxia in three human head and neck tumor lines. Radiother. Oncol. 2005, 76, 194–199. 10.1016/j.radonc.2005.06.031. 16024110

[ref43] Faes S.; Planche A.; Uldry E.; Santoro T.; Pythoud C.; Stehle J. C.; et al. Targeting carbonic anhydrase IX improves the anti-cancer efficacy of mTOR inhibitors. Oncotarget 2016, 7, 36666–36680. 10.18632/oncotarget.9134. 27153561 PMC5095030

[ref44] Hilvo M.; Rafajová M.; Pastoreková S.; Pastorek J.; Parkkila S. Expression of Carbonic Anhydrase IX in Mouse Tissues. J. Histochem. Cytochem. 2004, 52, 1313–1322. 10.1177/002215540405201007. 15385577

[ref45] Takacova M.; Barathova M.; Zatovicova M.; Golias T.; Kajanova I.; Jelenska L.; et al. Carbonic Anhydrase IX-Mouse versus Human. Int. J. Mol. Sci. 2020, 21, 246 10.3390/ijms21010246. PMC6982145 31905844

[ref46] Carriere V.; Colisson R.; Jiguet-Jiglaire C.; Bellard E.; Bouche G.; Al Saati T.; et al. Cancer cells regulate lymphocyte recruitment and leukocyte-endothelium interactions in the tumor-draining lymph node. Cancer Res. 2005, 65, 11639–11648. 10.1158/0008-5472.CAN-05-1190. 16357175

[ref47] Huizing F. J.; Garousi J.; Lok J.; Franssen G.; Hoeben B. A. W.; Frejd F. Y.; et al. CAIX-targeting radiotracers for hypoxia imaging in head and neck cancer models. Sci. Rep. 2019, 9, 18898 10.1038/s41598-019-54824-5. 31827111 PMC6906415

[ref48] Brooks J. M.; Menezes A. N.; Ibrahim M.; Archer L.; Lal N.; Bagnall C. J.; et al. Development and Validation of a Combined Hypoxia and Immune Prognostic Classifier for Head and Neck Cancer. Clin. Cancer Res. 2019, 25, 5315–5328. 10.1158/1078-0432.CCR-18-3314. 31182433

[ref49] Rühle A.; Grosu A. L.; Wiedenmann N.; Stoian R.; Haehl E.; Zamboglou C.; et al. Immunohistochemistry-based hypoxia-immune prognostic classifier for head-and-neck cancer patients undergoing chemoradiation - Post-hoc analysis from a prospective imaging trial. Radiother. Oncol. 2021, 159, 75–81. 10.1016/j.radonc.2021.03.014. 33753155

[ref50] Craig S. G.; Humphries M. P.; Alderdice M.; Bingham V.; Richman S. D.; Loughrey M. B.; et al. Immune status is prognostic for poor survival in colorectal cancer patients and is associated with tumour hypoxia. Br. J. Cancer 2020, 123, 1280–1288. 10.1038/s41416-020-0985-5. 32684627 PMC7555485

[ref51] Chafe S. C.; Riaz N.; Burugu S.; Gao D.; Leung S. C. Y.; Lee A. F.; et al. Granulocyte Colony Stimulating Factor Expression in Breast Cancer and Its Association with Carbonic Anhydrase IX and Immune Checkpoints. Cancers 2021, 13, 1022 10.3390/cancers13051022. 33804486 PMC7957699

[ref52] Koh Y. W.; Lee S. J.; Han J. H.; Haam S.; Jung J.; Lee H. W. PD-L1 protein expression in non-small-cell lung cancer and its relationship with the hypoxia-related signaling pathways: A study based on immunohistochemistry and RNA sequencing data. Lung Cancer 2019, 129, 41–47. 10.1016/j.lungcan.2019.01.004. 30797490

