# Supplementary material for: Quantitative Imaging of Hypoxic CAIX-Positive Tumor Areas with Low Immune Cell Infiltration in Syngeneic Mouse Tumor Models
Source: Mol Pharm. 2023 Mar 7;20(4):2245–55. doi: 10.1021/acs.molpharmaceut.3c00045 (PMC10074386; doi:10.1021/acs.molpharmaceut.3c00045)
Supplement: Supplementary file 1 — mp3c00045_si_001.pdf [file mp3c00045_si_001.pdf]

Supporting information: Quantitative imaging of hypoxic CAIX-positive tumor areas with low immune cell infiltration in syngeneic mouse tumor models

Daan F. Boreel[1,2], Paul N. Span[1], Annemarie Kip[2], Milou Boswinkel[2], Johannes P.W. Peters[1], Gosse J. Adema[1], Johan Bussink[1]*, Sandra Heskamp[2]*

[1]Radiotherapy and OncoImmunology Laboratory, Radiation Oncology, Radboud University Medical Center, Geert Grooteplein zuid 32, 6525GA, Nijmegen, the Netherlands.

[2]Department of Medical Imaging, Radboud University Medical Center, Geert Grooteplein zuid 10, 6525GA, Nijmegen, the Netherlands.

Corresponding author: Daan F. Boreel, PhD student, Geert Grooteplein zuid 32, 6525 GA, Nijmegen, the Netherlands, Tel: +31 (024) 361 4505, Fax: +31 (024) 363 5127, daan.boreel@radboudumc.nl

* Contributed equally

Supplementary figures

Dose (µg protein)	Tumor-to-muscle	Tumor-to-blood
3	32.1 ± 11.8	7.3 ± 2.3
10	37.0 ± 7.7	6.1 ± 1.4
30	35.7 ± 4.4	3.3 ± 0.8
100	25.7 ± 4.2	1.8 ± 0.3
Timepoint (hours)		
24	24.4 ± 3.5	1.6 ± 0.3
48	27.8 ± 4.3	2.1 ± 0.4
72	36.5 ± 7.1	3.3 ± 0.7
72 irrelevant IgG1	11.3 ± 2.5	0.7 ± 0.2

Supplementary table S1. tumor-to-background ratio for dose and time optimization in mice bearing B16ova tumors.

**Tracer uptake (%ID/g)**	**Antibody dose (72 h post injection)**				**Time point (30 µg antibody)**			
	3 µg	10 µg	30 µg	100 µg	24 h	48 h	72 h	72 h irrelevant IgG1
Blood	2.4 ± 0.5	3.9 ± 0.6	9.5 ± 2.9	16.6 ± 2.3	24.9 ± 0.6	16.8 ± 2.1	10.9 ± 2.4	18.4 ± 1.7
Muscle	0.6 ± 0.2	0.7 ± 0.2	0.8 ± 0.2	1.2 ± 0.2	1.6 ± 0.2	1.3 ± 0.1	1.0 ± 0.2	1.2 ± 0.2
Tumor	16.6 ± 3.0	23.5 ± 3.1	30.3 ± 9.4	29.4 ± 5.7	40.0 ± 7.6	34.9 ± 5.4	34.6 ± 5.8	13.4 ± 2.7
Lung	26.0 ± 3.1	30.6 ± 2.9	26.6 ± 3.4	17.3 ± 1.6	31.3 ± 3.2	36.0 ± 3.0	37.3 ± 4.3	12.7 ± 2.3
Liver	31.6 ± 2.1	23.7 ± 2.7	16.4 ± 2.2	8.8 ± 1.0	22.2 ± 0.6	28.7 ± 1.5	29.0 ± 1.4	3.9 ± 1.0
Kidney	5.0 ± 0.7	5.6 ± 0.5	6.3 ± 0.7	6.5 ± 0.9	11.5 ± 0.7	9.7 ± 0.5	8.2 ± 0.8	6.0 ± 0.5
Stomach	4.8 ± 1.7	6.2 ± 0.8	8.1 ± 2.0	12.0 ± 2.2	10.9 ± 1.2	10.0 ± 1.7	8.3 ± 1.0	2.0 ± 0.5
Duodenum	5.9 ± 1.2	10.8 ± 1.3	12.0 ± 2.3	11.1 ± 2.3	15.9 ± 1.5	13.4 ± 3.4	13.0 ± 3.9	3.2 ± 1.2
Jejunum	5.0 ± 1.2	7.5 ± 1.0	8.8 ± 2.4	7.9 ± 1.1	11.7 ± 2.8	10.7 ± 2.1	9.7 ± 2.4	2.9 ± 1.0
Ileum	3.7 ± 0.9	4.5 ± 0.8	5.6 ± 1.2	5.2 ± 0.9	10.5 ± 0.6	8.1 ± 1.4	6.5 ± 1.6	3.0 ± 1.2
Colon	2.1 ± 0.3	3.0 ± 0.5	4.3 ± 1.1	4.1 ± 0.6	6.0 ± 0.3	4.9 ± 0.2	3.7 ± 0.6	1.8 ± 0.5
Lymph nodes	2.7 ± 0.3	4.3 ± 0.9	5.1 ± 1.2	7.0 ± 1.1	8.4 ± 0.8	9.0 ± 1.0	8.1 ± 1.3	8.8 ± 2.3
Brown fat	2.8 ± 0.5	2.8 ± 0.3	3.3 ± 0.5	4.3 ± 0.7	5.7 ± 0.1	5.9 ± 0.3	4.0 ± 0.2	4.1 ± 0.9
Thymus	1.3 ± 0.1	1.8 ± 0.4	2.4 ± 0.4	3.5 ± 0.7	4.8 ± 0.7	4.4 ± 0.5	3.1 ± 0.3	3.9 ± 0.2
Heart	1.7 ± 0.3	2.0 ± 0.2	2.6 ± 0.5	4.0 ± 0.9	6.8 ± 0.6	5.2 ± 0.4	3.7 ± 0.3	4.4 ± 0.5
Spleen	7.8 ± 1.1	6.9 ± 0.9	6.1 ± 0.7	5.7 ± 1.0	11.0 ± 0.7	11.6 ± 0.4	11.5 ± 0.8	5.0 ± 0.6
Pancreas	3.0 ± 0.6	3.8 ± 0.7	4.1 ± 0.8	4.1 ± 0.7	5.3 ± 0.7	4.2 ± 0.4	3.8 ± 0.4	2.3 ± 0.7
Bone-marrow	2.4 ± 0.2	3.0 ± 0.6	3.1 ± 0.6	5.3 ± 1.2	9.2 ± 0.7	8.0 ± 0.8	7.1 ± 0.7	7.3 ± 0.6
Bone	0.9 ± 0.1	1.0 ± 0.2	1.2 ± 0.2	1.9 ± 0.5	2.4 ± 0.1	2.2 ± 0.3	1.6 ± 0.1	1.7 ± 0.2

Supplementary table S2. Dose and Time optimization biodistribution data of [^{111}In]In-mCAIX and [^{111}In]In-hIgG1 in B16ova tumors and selected normal tissues.

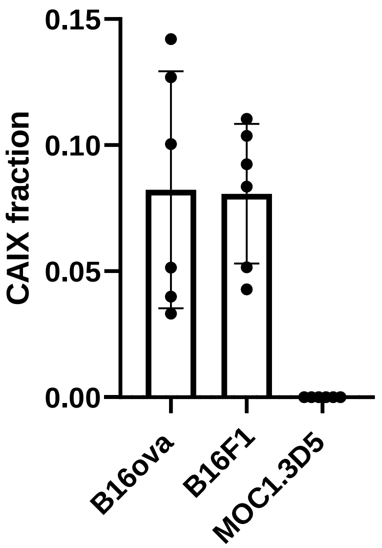

Supplementary figure S1. Fraction CAIX⁺ area in several tumor models analyzed by IHC.

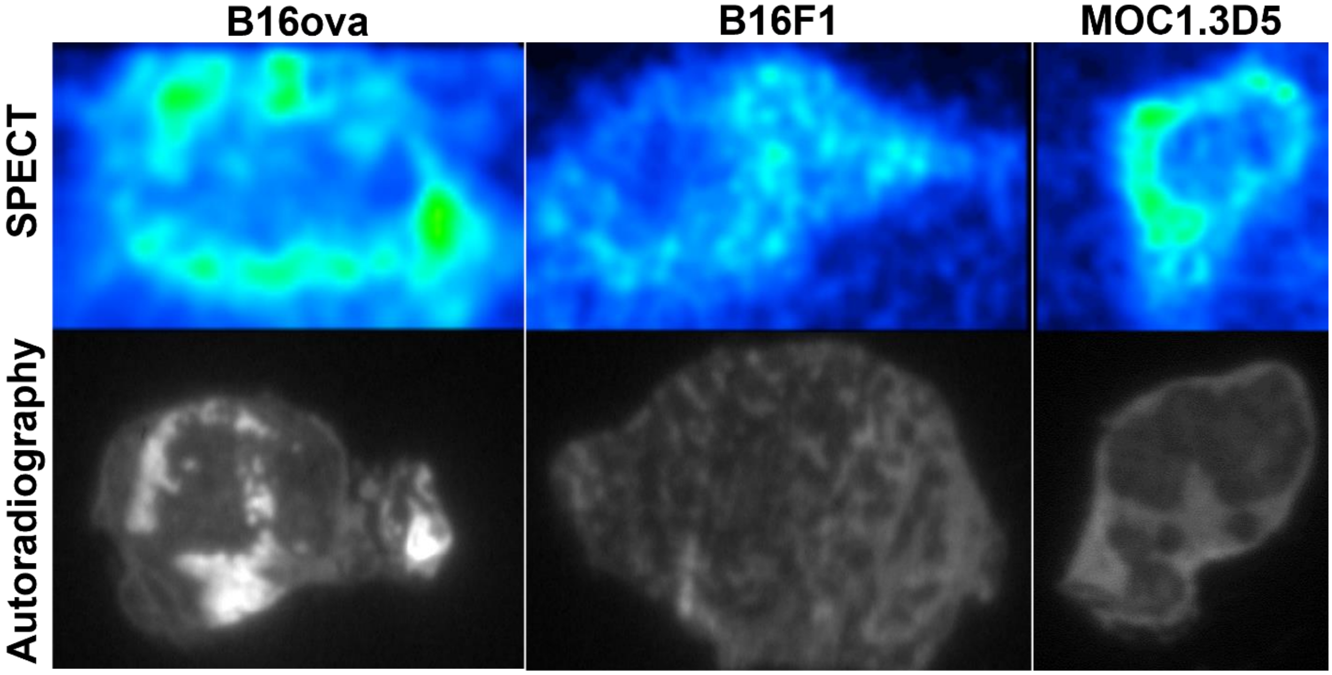

Supplementary figure S2. Cross-sections of microSPECT and AR images matched approximately.

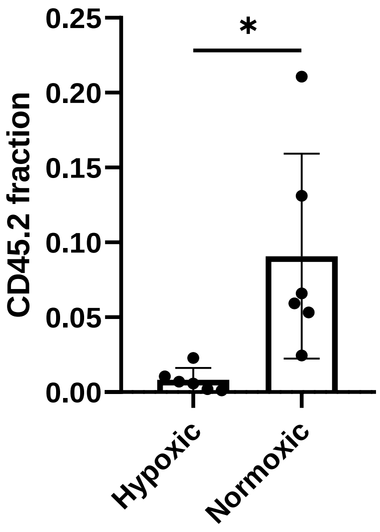

Supplementary figure S3. CD45.2 fraction in hypoxic (pimonidazole⁺) and normoxic (pimonidazole⁻) tumor areas of MOC1.3D5 whole tumor sections, n=6.
